# Emerging Role of Gut Microbiota in Breast Cancer Development and Its Implications in Treatment

**DOI:** 10.3390/metabo14120683

**Published:** 2024-12-05

**Authors:** Rashid Mir, Shrooq A. Albarqi, Wed Albalawi, Hanan E. Alatwi, Marfat Alatawy, Ruqaiah I. Bedaiwi, Reema Almotairi, Eram Husain, Mohammad Zubair, Ghaida Alanazi, Shouq S. Alsubaie, Razan I. Alghabban, Khalid A. Alfifi, Shabnam Bashir

**Affiliations:** 1 Department of Medical Lab Technology, Faculty of Applied Medical Sciences, Prince Fahd Bin Sultan Research Chair for Biomedical Research, University of Tabuk, Tabuk 47713, Saudi Arabia; rbedaiwi@ut.edu.sa (R.I.B.); ralmotairi@ut.edu.sa (R.A.); e.husain@ut.edu.sa (E.H.); 2 Molecular Medicine, Department of Medical Lab Technology, Faculty of Applied Medical Sciences, University of Tabuk, Tabuk 47713, Saudi Arabia; 441000436@stu.ut.edu.sa (S.A.A.); 441000437@stu.ut.edu.sa (W.A.); 441000370@stu.ut.edu.sa (G.A.); 441000379@stu.ut.edu.sa (S.S.A.); 441000348@stu.ut.edu.sa (R.I.A.); 3 Department of Biology, Faculty of Science, University of Tabuk, Tabuk 47713, Saudi Arabia; h_alatwi@ut.edu.sa (H.E.A.); mevalatawi@ut.edu.sa (M.A.); 4 Department of Medical Microbiology, Faculty of Medicine, University of Tabuk, Tabuk 47713, Saudi Arabia; zmohammad@ut.edu.sa; 5 Department of Laboratory and Blood Bank, King Fahd Special Hospital, Tabuk 47717, Saudi Arabia; khalfifi@moh.gov.sa; 6 Mubarak Hospital, Srinagar 190002, Jammu and Kashmir, India; shabnam.bashir@gmail.com

**Keywords:** gut microbiota, breast cancer, dysbiosis, hormone metabolism, cancer immunotherapy

## Abstract

**Background:** The human digestive system contains approximately 100 trillion bacteria. The gut microbiota is an emerging field of research that is associated with specific biological processes in many diseases, including cardiovascular disease, obesity, diabetes, brain disease, rheumatoid arthritis, and cancer. Emerging evidence indicates that the gut microbiota affects the response to anticancer therapies by modulating the host immune system. Recent studies have explained a high correlation between the gut microbiota and breast cancer: dysbiosis in breast cancer may regulate the systemic inflammatory response, hormone metabolism, immune response, and the tumor microenvironment. Some of the gut bacteria are related to estrogen metabolism, which may increase or decrease the risk of breast cancer by changing the number of hormones. Further, the gut microbiota has been seen to modulate the immune system in respect of its ability to protect against and treat cancers, with a specific focus on hormone receptor-positive breast cancer. Probiotics and other therapies claiming to control the gut microbiome by bacterial means might be useful in the prevention, or even in the treatment, of breast cancer. **Conclusions:** The present review underlines the various aspects of gut microbiota in breast cancer risk and its clinical application, warranting research on individualized microbiome-modulated therapeutic approaches to breast cancer treatment.

## 1. Introduction

The microbiota, a vast community of trillions of bacteria, inhabits the human body, creating a complex ecology [1]. These microorganisms, which are made up of bacteria, viruses, fungus, and protozoa, live in different parts of the body, including the mouth, skin, and gastrointestinal system, where they create unique communities. Of these, the gut microbiota has become one of the most important, impacting several physiological functions and being essential for preserving health [2]. Our understanding of the gut microbiota has transformed over the past 20 years due to developments in molecular biology and next-generation sequencing technologies, which have shown how intricately it is involved in everything from metabolism and immunological function to mental health and illness [3].

As the most common cancer in women diagnosed globally, breast cancer is a complex and multifaceted illness that continues to be a major cause of cancer-related mortality [4]. Despite extensive research, the etiology of breast cancer remains incompletely understood, with known genetic and environmental risk factors explaining only a fraction of cases. Conventional risk factors for breast cancer consist of age, family history, hormones, and lifestyle decisions including food and exercise [5]. However, a significant proportion of breast cancer cases occur in women who do not exhibit any of these established risk factors, suggesting that there are additional, as yet unidentified, contributors to the disease [4,6]. In recent years, the gut microbiota has emerged as a novel and intriguing player in the development and progression of breast cancer [7]. The concept that the microorganisms residing in the gut could influence cancer development in a distant organ such as the breast might seem counterintuitive at first [8]. However, growing evidence suggests that the gut microbiota may significantly impact breast cancer risk through various mechanisms, including modulation of systemic inflammation, hormone metabolism, immune response, and even direct effects on the tumor microenvironment [9].

**Figure 1:**
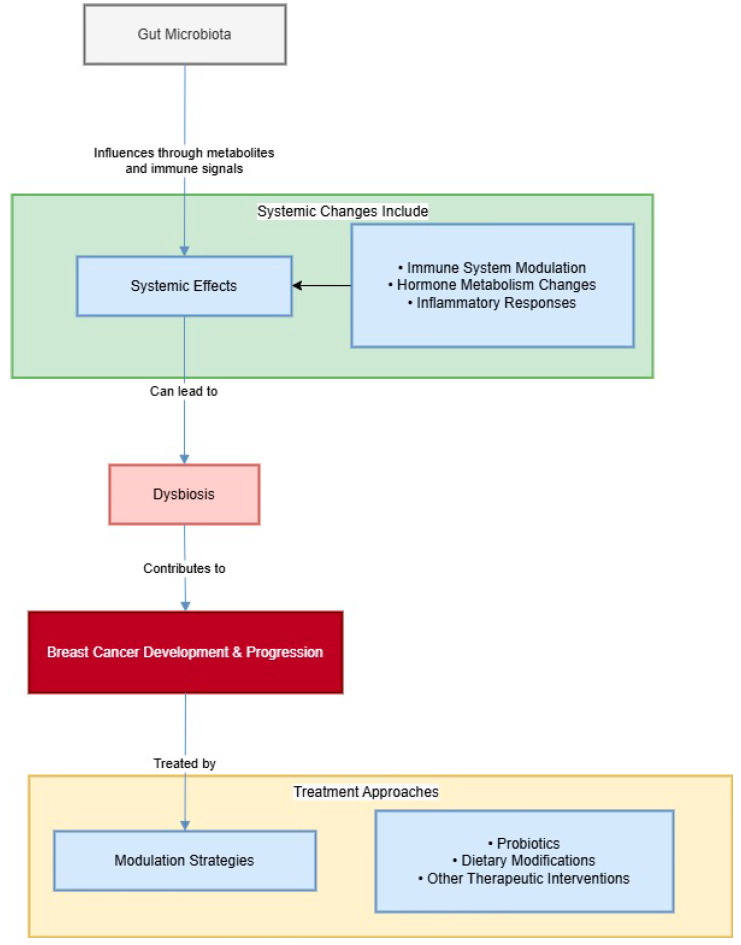
The biological events are presented in the diagram which demonstrates how gut microbiota affects breast cancer. It begins with gut microbiota initiating catabolic processes throughout the body with emphases on the immune system and hormones. These systemic changes can then result in dysbiosis which is the disturbance of the normal bacterial flora in the gut. Dysbiosis is known to cause and aggravate the process of breast cancer when present in the body. Finally, the diagram also indicates that we can influence this process in some ways using modulation strategies such as the use of probiotics, changes in the diet and other therapeutic measures that are directed at the restoration of the needed and desired balance in the gut as well as the prevention or treatment of breast cancer.

The immune system of the host and other physiological functions are constantly interacting with the dynamic and complex population known as the gut microbiota [10]. An imbalance in the makeup and activity of the gut microbiota, or dysbiosis, has been linked to a number of illnesses, including colorectal cancer, obesity, diabetes, and inflammatory bowel disease [11]. Similarly, emerging research suggests that dysbiosis might also contribute to the development and progression of breast cancer [12]. For instance, certain gut bacteria have been shown to metabolize estrogens, potentially altering systemic hormone levels and influencing breast cancer risk [13]. Additionally, the gut microbiota can modulate immune responses, which are critical in both cancer development and the efficacy of cancer therapies [14,15]. To better understand these connections, Figure 1 illustrates the stepwise interactions between the gut microbiota and breast cancer, along with potential intervention strategies aimed at restoring microbiota balance and enhancing therapeutic outcomes.

There is a strong theory that breast cancer may be prevented or treated by altering the gut flora. In the context of cancer treatment, a number of methods for altering the gut microbiota are now being investigated, including dietary modifications, probiotics, prebiotics, antibiotics, and fecal microbiota transplantation [16,17,18]. Through these interventions, the immune system will be strengthened, inflammation will be decreased, and the efficacy of traditional cancer treatments like chemotherapy and immunotherapy will be increased [19,20].

One of the most promising areas of research is the interaction between the gut microbiota and the immune system in the context of cancer. The immune system plays a dual role in cancer, acting as both a protector against tumor formation [21] and a promoter of tumor progression under certain conditions [22]. There is growing evidence that some gut bacteria can modify the immune response to cancer. The gut microbiota is known to affect immune cell growth and function [23,24,25].

In addition to its effects on the immune system, the gut microbiota may also influence breast cancer through its role in metabolizing dietary components and drugs [26]. The gut microbiota is involved in the metabolism of various compounds, including carcinogens, hormones, and drugs [27]. By modifying the bioavailability and activity of these compounds, the gut microbiota can potentially impact cancer risk and treatment outcomes [28]. Phytoestrogens are plant-derived chemicals that, depending on the situation, can have both estrogenic and anti-estrogenic effects. Certain gut bacteria are able to digest these molecules [29]. The interaction between dietary components, the gut microbiota, and breast cancer risk is an area of active research, with the potential to uncover new dietary strategies for cancer prevention and treatment.

Furthermore, the gut microbiota’s influence extends beyond the gut, affecting distant organs through the release of metabolites, microbial components, and signaling molecules into the bloodstream [30]. These compounds have the ability to penetrate the tumor microenvironment and breast tissue, where they may have a variety of effects, such as stimulating or impeding the development of the tumor. For example, it has been demonstrated that short-chain fatty acids (SCFAs), which are created when gut bacteria digest dietary fiber, have anti-inflammatory and anti-cancer qualities [31]. On the other hand, certain bacterial toxins and metabolites may promote tumorigenesis by causing DNA damage or modulating cell-signaling pathways [32].

The direct effects of gut bacteria on the tumor are not the only way that the gut microbiota and breast cancer are related. Additionally, there are data that suggest the microbiome found in breast tissue may be unique and might contribute to the development of cancer [7,33,34]. While the breast was traditionally considered a sterile environment, recent studies have identified a diverse community of bacteria within breast tissue, with differences in microbial composition between healthy and cancerous tissues [35,36,37,38,39]. These results highlight significant concerns regarding the possible roles of the gut microbiota in affecting tumor behavior, modifying the local immune response, and altering the risk of breast cancer through interactions with it.

A one-size-fits-all strategy for altering the microbiome for cancer prevention and therapy is obviously unlikely to be successful given the complexity of the gut microbiota and its interactions with the host. Instead, personalized strategies that take into account an individual’s unique microbiota composition, genetic background, diet, and lifestyle are likely to be more successful. Technological developments in metagenomics, metabolomics, and systems biology are enabling the creation of customized strategies that have the potential to completely transform cancer therapy and prevention.

## 2. Bridging the Gap: Linking Gut Microbes to Breast Health

According to a 2018 worldwide research, microbial infections—including both bacterial and viral infections—account for 13% of the world’s cancer burden, with a pronounced geographic correlation [40,41]. *Hepatitis B* virus (*HBV*), *Helicobacter pylori* (*H. pylori*), *Human papillomavirus* (*HPV*), and *Hepatitis C* virus (*HCV*) were the main causal agents that were found [41]. Remarkably, 10–100 trillion microbial partners make up the human microbiome, the majority of which are yet unknown. Eastern Asia had the greatest rates of infection-related cancer development, followed by Sub-Saharan Africa, Northern Europe, and West Asia. Specifically, one-third of the malignancies caused by *HPV* and *H. pylori* were found in China [41].

The significance of examining the function of microorganisms in cancer, particularly breast cancer, is highlighted by this study. Breast cancer is actually a group of distinct breast cancers, each with a distinct molecular subtype, rather than a unified illness. Based on the existence or lack of cell surface receptors, including HER2, progesterone receptors (PRs), and estrogen receptors (ERs), several subtypes have been identified. Due to the paucity of targeted medicines, triple-negative breast cancer (TNBC), which lacks all three receptors, is extremely difficult to treat and is linked to worse outcomes [42].

Breast cancer is a complex illness that is impacted by a number of variables, including genetics, age, lifestyle, parity, and exposure to carcinogens [43]. Apart from being a woman and being older than 50, 70% of breast cancers are discovered without any established risk factors. Over the past forty years, the incidence of breast cancer has risen by 0.5% yearly despite breakthroughs in early diagnosis, contemporary treatment approaches, and increasing public awareness; nonetheless, the death rate has dramatically decreased [44]. Still, there is a lack of consistency in forecasting results, especially for younger and less advantaged women, indicating the presence of other determinants. The emergence of contemporary sequencing technology and multicentric approaches has drawn focus to the microbiome as a putative predictor of the severity and mortality of breast cancer [45]. The gut microbiota and local breast microbiome can have a favorable or unfavorable effect on the risk of breast cancer. By controlling levels of circulating steroid hormones, energy intake and utilization, and the synthesis of compounds such as genotoxins, the microbiota may have an impact on breast cancer [40]. To better understand the microbial contribution to cancer development, Table 1 highlights key microbial infections and their association with cancer across different regions.

## 3. Microbial Machinations: How Gut Bacteria Influence Breast Cancer

**Figure 2:**
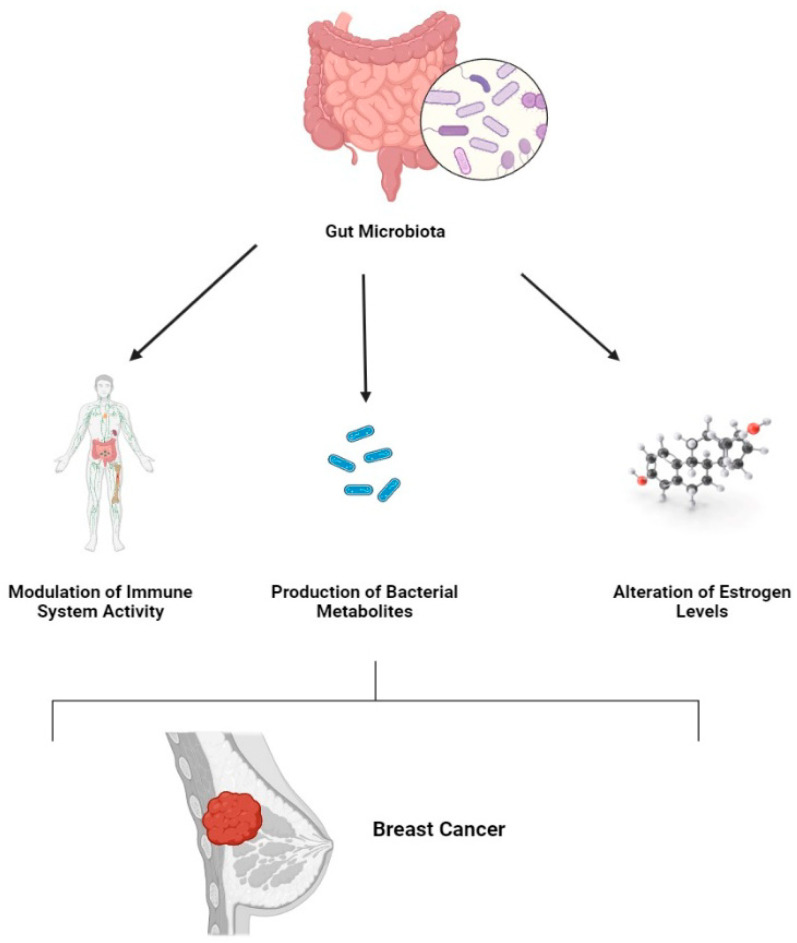
Breast cancer and the influence of gut microbiota. This figure depicts the role of gut microbiota in breast cancer progression, highlighting mechanisms such as immune modulation, estrogen level alteration, and the production of bacterial metabolites.

One of the main causes of cancer-related mortality in women worldwide is breast cancer. It is not a single disease but rather a collection of different subtypes, each with its own characteristics, prognosis, and response to treatment. Luminal A, triple-negative breast tumors (TNBCs), HER2-positive, and Luminal B are some of these subtypes [51]. Treatment and prognosis for breast cancer have historically been determined by the presence or lack of particular molecular markers, such as the HER2 protein, progesterone receptors (PR), and estrogen receptors (ER) [52,53,54]. Nonetheless, current studies indicate that the large population of bacteria in our digestive systems, known as the gut microbiome, may potentially be a major factor in the onset and spread of breast cancer [7,8,55,56] (Figure 2).

### 3.1. Immune Interplay: Gut Microbes as Modulators of Cancer Defense

The gut microbiome is essential for regulating our immune system, helping to maintain a balance between the body and the bacteria that live within it [57]. Currently, one of the most well-defined ways in which the gut microbiome impacts the systemic immune response is through its effect on the T cell compartment of the adaptive immune system. Studies have shown that gut microbiota can influence the differentiation of T cell populations into various subtypes, including T-helper (Th) cells such as Th1, Th2, and Th17, as well as regulatory T cells (Tregs) [58,59]. Beyond their role in shaping T cell development and function, signals derived from gut microbiota also modulate innate immune defenses. These microbial signals stimulate lymphoid tissues in the spleen, regulate the neutrophil migration and function, induce and activate macrophages, and enhance the maturation and functionality of natural killer (NK) cells [60,61]. More recently, specific bacterial species have been shown to influence inflammatory responses by reducing plasma corticosterone levels—an anti-inflammatory steroid that plays a key role in managing inflammation following mucosal injury [62].

In studies involving hormone receptor-positive (HR+) breast cancer (where tumors grow in response to hormones such as estrogen), it was observed that when the normal gut bacteria were disrupted by antibiotics, there was an increase in inflammatory cells in the tumor [63,64,65]. These cells, particularly a type called M2-like macrophages, are often found in higher numbers in breast cancer patients with poorer outcomes [66,67]. This research underscores how an unhealthy gut microbiome can impact the immune system in ways that support breast cancer progression.

### 3.2. Microbiota’s Role in Estrogen Regulation

Hormones such as estrogen are important in the development of hormone receptor-positive breast cancer [68]. The gut microbiota, especially a subset called the estrobolome, plays a crucial role in estrogen metabolism [69]. After the liver modifies estrogen for excretion, gut bacteria with enzymes like β-glucuronidase can deconjugate it, allowing estrogen to be reabsorbed into the bloodstream—a process called enterohepatic recirculation [70]. This recycling affects estrogen levels in the body, impacting hormonal balance and disease risk. An imbalance in gut bacteria (dysbiosis) can disrupt this process, potentially increasing the risk of hormone-related conditions such as breast cancer [71]. Diet, lifestyle, and probiotics all influence the gut microbiota and, consequently, estrogen metabolism [63,72].

**Figure 3:**
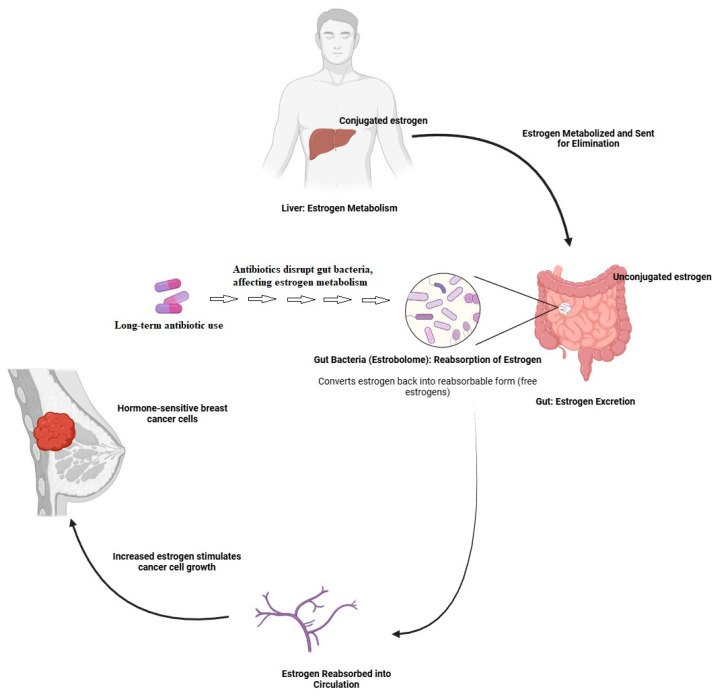
Interaction of gut microbiota and estrogen metabolism in breast cancer risk. Illustration of estrogen metabolism by the liver, the role of gut microbiota (estrobolome) in estrogen reabsorption, and its impact on hormone-sensitive breast cancer risk.

Because increased amounts of estrogen can drive the growth of hormone-sensitive breast cancer cells, this reabsorption process can raise the risk of breast cancer formation and progression [73,74]. The “estrobolome” is the name used to describe this relationship between gut bacteria and estrogen levels [75]. Long-term antibiotic usage has been linked to disruption of the gut microbiota and an increased risk of breast cancer, according to studies [76,77,78] (Figure 3).

Hormone therapy, commonly referred to as endocrine therapy, is a cornerstone in the treatment of hormone-sensitive breast cancer [79]. This approach targets cancers that are influenced by hormones such as estrogen and progesterone. Although research on the connection between hormone therapy and the gut microbiome in breast cancer is still ongoing, early results point to the possibility that the gut microbiota may influence how these therapies work [80].

Given the enormous effect of gut bacteria on the body’s estrogen levels, estrogen metabolism is an important field of study. β-glucuronidase, an enzyme that breaks down estrogens into their active forms, is one way that the gut microbiota helps regulate estrogen levels [81]. The balance of hormones is maintained by this mechanism. A change in this mechanism, commonly owing to gut dysbiosis, can lead to altered estrogen levels, which may influence hormone-sensitive malignancies like breast cancer [72,75,82,83].

Studies have indicated that changes in the gut microbiota, influenced by factors such as food, ethnicity, and antibiotic exposure, might affect estrogen metabolism and, subsequently, breast cancer risk [80]. The consumption of soy products, which contain phytoestrogens, has been linked to a reduced risk of breast cancer in some studies [84,85]. Probiotics, such as Lactobacillus casei Shirota, have also shown potential in reducing breast cancer incidence, possibly through their interaction with estrogen metabolism [86,87] (Table 2).

### 3.3. Bacterial By-Products in Cancer Progression

The gut microbiome also produces various substances, known as metabolites, that can impact breast cancer. It has been discovered that some gut bacteria create a chemical known as lithocholic acid (LCA), which inhibits the growth of breast cancer cells [92,93]. LCA can also trigger immune responses that help the body fight cancer [94]. Short-chain fatty acids (SCFAs) are created when gut bacteria break down food fibers, including butyrate. SCFAs are another significant class of metabolites [31,95]. Particularly, butyrate has demonstrated potential in reducing the proliferation of breast cancer cells and improving the efficacy of cancer therapies [96,97]. These findings suggest that some toxins (like Cytolethal distending toxin (CDT) and colibactin) produced by gut bacteria can have a significant impact on breast cancer, either by slowing its growth or by helping the immune system to fight it more effectively (Table 3).

The gut microbiome exerts a broad impact on breast cancer risk beyond its localized effects on breast tissue. It influences overall cancer susceptibility by modulating hormone levels, energy metabolism, and immune function [40,98]. The potential significance of the gut microbiome in the development and management of breast cancer is indicated by its capacity to generate diverse metabolites and regulate the immune system. The significance of the gut microbiome in breast cancer is highlighted by this new research, which also suggests that patients may benefit from novel therapies that target gut bacteria. Scientists and medical professionals may be able to create more potent preventative and therapeutic approaches to breast cancer by comprehending the intricate relationships between the gut microbiota and the illness.

**Table 3:** Overview of gut microbiome mechanisms influencing breast cancer progression.

Gut Microbiome Aspect	Mechanism	Impact on Breast Cancer	Reference
Immune Modulation	Through M2-like macrophages, dysbiosis increases inflammation and encourages the formation of cancer.	Encourages the growth of tumors, especially in HR+ breast cancer.	[7]
Estrogen Regulation (Estrobolome)	Estrogens intended for excretion are reabsorbed by gut flora.	The risk of hormone-sensitive breast cancers is increased by higher amounts of estrogen.	[69]
Bacterial By-products (LCA)	Lithocholic acid (LCA) inhibits the growth of breast cancer cells and stimulates the immune system.	Boosts immune function and slows the spread of cancer.	[99]
Bacterial By-products (SCFAs)	SCFAs, particularly butyrate, reduce the development of cancer cells and enhance the effectiveness of therapies.	Possible therapeutic benefit in slowing the proliferation of malignant cells	[100]
Systemic Effects	Affects immunological response, hormone levels, and energy metabolism	Extensive effects on cancer risk and progression and the possibility of novel treatment modalities	[3]

## 4. The Breast’s Own Microbial Community and Its Role in Breast Cancer Development

The significance of the breast microbiome in the onset and spread of breast cancer has been highlighted by recent studies. It has now been discovered that the breast tissue, until recently believed to be sterile, is home to a wide variety of bacteria. These microorganisms, primarily bacteria, are increasingly recognized for their influence on cancer biology. However, the origins of these bacteria and their precise roles in breast cancer remain areas of active investigation. The below section explores the current understanding of the breast microbiome, its potential origins, its alterations in cancer, and the mechanisms through which it may affect tumor development and progression.

### 4.1. Microbial Origins

The question of how bacteria come to reside in the breast tissue is complex and multifaceted. One hypothesis suggests that bacteria may translocate from the oral cavity to the breast tissue. This idea is supported by studies showing the presence of *Bacteroides* species in both canine breast tumors and the oral cavity, implying a potential route from the mouth to the mammary tissue via the gut. This pathway is thought to involve the migration of bacteria from the gut into the bloodstream and subsequently to the breast tissue [12,101,102].

Another hypothesis posits that bacteria found in the breast tissue may originate from the skin. Evidence supporting this comes from the identification of *Staphylococcus epidermidis* and *Micrococcus luteus*, which are typically skin-resident bacteria, in mammary tumors. These microbes may enter the breast tissue through the nipples and spread throughout the glandular structures [103,104,105]. This scenario highlights the possibility that the mammary microbiome may be influenced by external microbial sources. Recent studies have also identified live bacteria within both tumor and tumor-associated immune cells [106,107,108]. This observation suggests that cancer cells and immune cells may serve as vehicles for the dissemination of bacteria within the breast tissue. The presence of bacteria in such locations underscores a dynamic interaction between microbial and host cells that could facilitate microbial colonization and potentially influence cancer development.

Another area of ongoing investigation is the relationship between breast cancer and the gut flora. Toxins known to be pro-carcinogenic produced by gut bacteria include colibactin and BFT, which may enter the circulation and end up in breast tissue [40]. Additionally, gut microbes can produce metabolites that affect breast cancer progression. For instance, gut-derived estrogens and estrogen mimics can influence the levels of active estrogens in circulation, impacting breast cancer risk [40].

**Figure 4:**
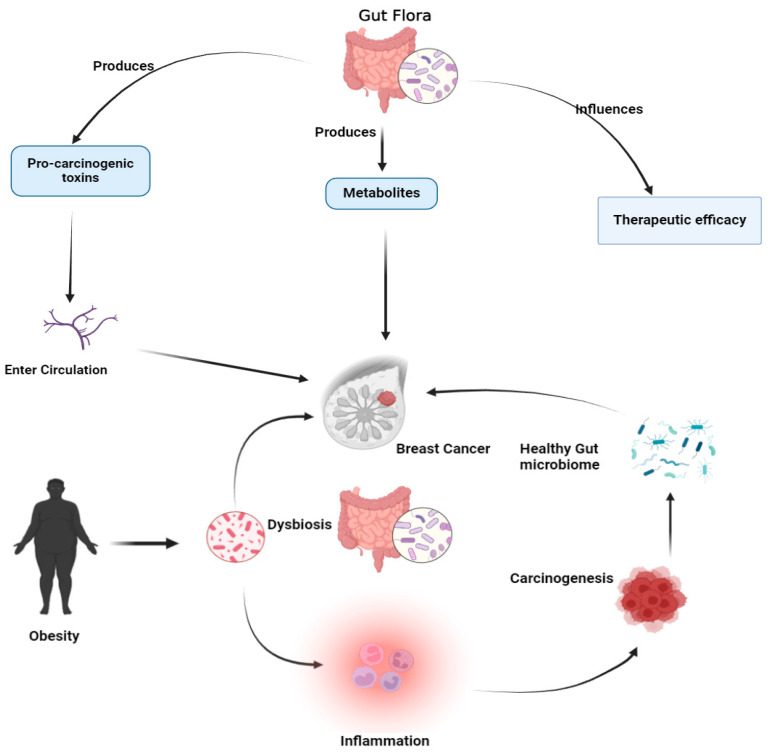
Gut flora and breast cancer interactions. This diagram illustrates the complex relationship between gut microbiota and breast cancer, showing how gut flora influences cancer development, progression, and treatment through various mechanisms.

Additionally, the gut microbiota regulates inflammation and immunological responses, both of which are essential for the development of cancer. An imbalance in the gut microbiota known as dysbiosis has been connected to a number of illnesses, including breast cancer. Variations in the variety of gut microbes can influence the immune system’s capacity to combat cancer cells and cause systemic inflammation [109]. The interaction between the microbiome and cancer is further complicated by the association between obesity and gut dysbiosis, which is a substantial risk factor for breast cancer [110] (Figure 4).

Furthermore, the efficient use of therapeutic therapies depends on a healthy gut microbiota. Studies have demonstrated that the gut microbiota can affect the effectiveness of medications, such as immunotherapies and chemotherapeutics, underscoring the necessity of a thorough comprehension of microbiome interactions in cancer therapy [111].

It has long been known that the gut microbiota influences several physiological functions, such as metabolism, immunological response, and digestion, and that these activities contribute to general health maintenance [112]. Interesting relationships between the gut microbiota and other bodily systems, such as the liver, lungs, and brain, have been shown by recent studies [113,114]. It has not been proven beyond doubt that there is a direct connection between the mammary glands and the gut flora. In spite of this, strong data point to a possible connection between breast and intestinal health, raising the possibility of a complicated and maybe important interaction.

### 4.2. Potential Pathways of Gut–Breast Microbiota Interaction

The movement of bacteria that live in the gut is one conceivable way that the gut microbiota and breast tissue interact [55]. Breaches in the intestinal epithelium can happen when there is dysbiosis, which is defined as an imbalance in the gut microbiota. These breaches may allow bacteria to escape the gut and enter the bloodstream or lymphatic system, potentially reaching the mammary glands [115]. Alternatively, intestinal dendritic cells, which play a crucial role in immune surveillance, could capture bacteria in the gut. These dendritic cells can then migrate to distant sites, including the mammary tissue, via the vascular system [116].

Another factor in this potential gut–breast axis could be bacterial metabolites. Produced by gut bacteria, these metabolites might be absorbed into the bloodstream through the intestinal mucosa and distributed throughout the body, including the mammary glands. The biological functions of these metabolites could potentially influence breast tissue health and disease [117].

Despite these theoretical pathways, concrete evidence confirming the existence of a gut–breast microbiota axis remains scarce. Further research is necessary to substantiate these connections and to elucidate the specific microbial and metabolic players involved in this potential crosstalk.

### 4.3. Microbial Changes in Cancerous Breast Tissue

**Figure 5:**
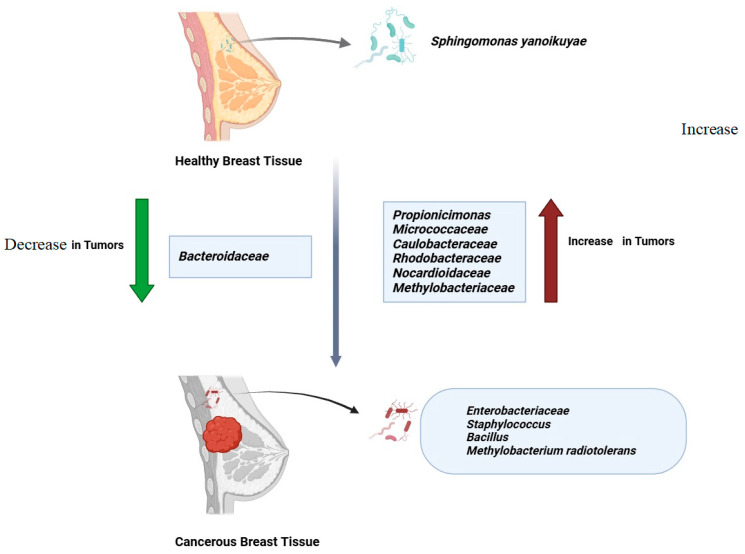
Changes in breast microbiome composition in response to cancer. This diagram illustrates the differences in microbial composition between healthy and cancerous breast tissues, highlighting the specific bacteria that increase or decrease in response to breast cancer.

The breast microbiome is not static; it changes in response to the presence of cancer. Numerous studies have documented shifts in microbial composition between normal and cancerous breast tissues [118,119] (Figure 5).

When compared to healthy persons, patients with breast cancer frequently have larger abundances of *Enterobacteriaceae*, *Staphylococcus*, and *Bacillus* [103,120]. Conversely, *Sphingomonas yanoikuyae*, typically present in normal breast tissue, is significantly reduced in tumor tissues, while *Methylobacterium radiotolerans* is notably enriched in tumors [7,64]. These microbial changes are not uniform across different cancer stages or subtypes. For example, research on an Asian cohort revealed that *Propionicimonas*, *Micrococcaceae*, *Caulobacteraceae*, *Rhodobacteraceae*, *Nocardioidaceae*, and *Methylobacteriaceae* are enriched in breast tumors, whereas *Bacteroidaceae* decreases and *Agrococcus* increases as malignancy progresses [121,122,123]. Similarly, variations in microbiome composition have been linked to different breast cancer molecular subtypes, including estrogen receptor-positive (ER+), triple-negative breast cancer (TNBC), and HER2-positive subtypes [121,124,125].

### 4.4. Mechanisms of Microbiome Influence on Breast Cancer

**Figure 6:**
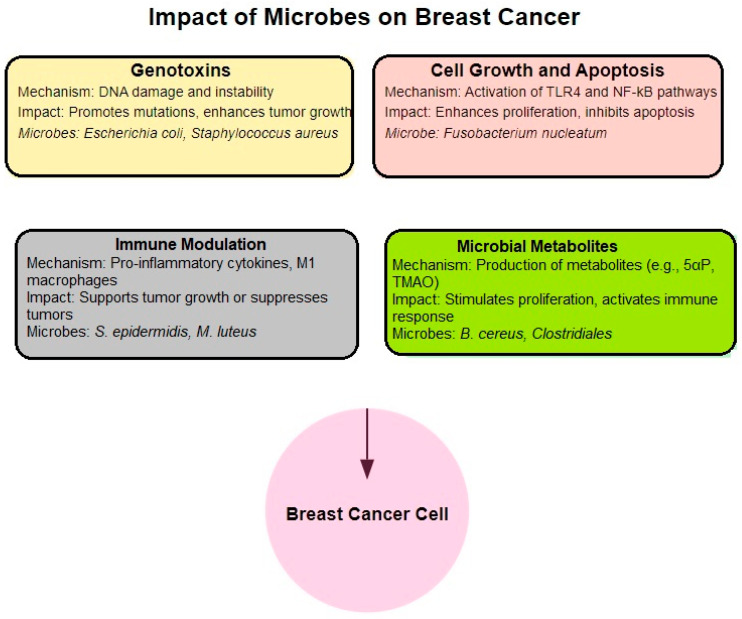
Key mechanisms of the breast microbiome’s influence on breast cancer development and progression.

The impact of the breast microbiome on cancer progression can be mediated through several mechanisms, including direct carcinogenic effects, modulation of cell growth and apoptosis, effects on immune responses, and the production of microbial metabolites (Figure 6).

Recent research has shed light on the complex relationship between the mammary microbiota and breast cancer progression. Studies have identified several bacterial species associated with breast cancer tissue in both human patients and murine models. In human tissue, *Escherichia coli* and *Staphylococcus* have been shown to induce DNA double-strand breaks and genomic instability in vitro, potentially contributing to cancer development [103]. Interestingly, the *Clostridiales* species may inhibit tumor growth by producing trimethylamine N-oxide (TMAO), which activates CD8+ T cell-mediated antitumor immunity [126]. *Fusobacterium nucleatum* has been linked to breast tumor progression and metastasis through fap-2 dependent binding to Gal-GalNac in breast cancer tissue [127]. In mice, *Staphylococcus, Lactobacillus,* and *Streptococcus* species have been associated with breast tumor lung metastases by modulating stress responses and altering cancer cell viability and cytoskeleton [128]. Additionally, *Staphylococcus epidermidis* has been found to increase T regulatory cell infiltration in tumors and activate the complement pathway [7]. *Micrococcus luteus* has demonstrated potential anti-tumor effects by reducing mammary tumor growth and promoting an anti-tumoral M1 macrophage phenotype, while *Bacteroides fragilis* has been associated with breast tumor progression and metastasis via secretion of *B. fragilis* toxin (BFT) [7,101]. These findings highlight the diverse effects of the mammary microbiota on breast cancer, including interference with cellular pathways, DNA damage induction, immune system modulation, and the release of bacterial metabolites, emphasizing the potential importance of the microbiome in breast cancer progression and treatment.

#### 4.4.1. Carcinogenic Effects

Certain bacteria found in breast tumors have been shown to possess carcinogenic properties. *Escherichia coli* and *Staphylococcus aureus* produce genotoxins such as colibactin and other molecules that induce DNA double-strand breaks and genomic instability. These bacterial products can directly damage the host DNA, potentially leading to mutations that drive cancer development [101,129]. Furthermore, Bacteroides fragilis generates a toxin that stimulates the β-catenin–Notch1 signaling pathway, hence promoting tumor development and metastasis [130].

#### 4.4.2. Impact on Cell Growth and Apoptosis

Microbial interactions with host cells often involve pattern-recognition receptors (PRRs) such as Toll-like receptors (TLRs), which are critical for detecting microbial components and modulating immune responses [131]. In breast cancer, bacteria like *Fusobacterium nucleatum* have been implicated in tumor growth via the activation of TLR4 and the NF-kB pathway. This activation can enhance cancer cell proliferation and inhibit apoptosis. Furthermore, the binding of *Fusobacterium* to specific sugars on cancer cells accelerates tumor growth and metastasis [132,133].

#### 4.4.3. Modulation of Immune Responses

The breast microbiome can significantly influence the immune environment within tumors. *Staphylococcus epidermidis* has been shown to induce a highly inflammatory tumor microenvironment by stimulating the secretion of pro-inflammatory cytokines and activation of the complement pathway. This inflammation can support tumor growth and promote the accumulation of immunosuppressive T regulatory cells [102,124,134]. On the other hand, *Micrococcus luteus* has an anti-tumoral effect by promoting an M1 macrophage phenotype, which is associated with tumor suppression [135].

The bacterium *Sphingomonas*, prevalent in healthy breast tissue, has been linked to enhanced immune surveillance [136]. It activates invariant NKT cells, which are critical for controlling cancer metastases [137]. Increased levels of *Sphingomonas* in healthy tissue correlate with a higher expression of TLRs and antimicrobial response effectors, suggesting its role in sustaining a protective immune environment [138].

#### 4.4.4. Microbial Metabolites

Microbes in the breast tissue may also produce metabolites that affect tumor biology. *Bacillus cereus* has been found to metabolize progesterone into 5-alpha-pregnane-3,20-dione (5αP), a compound that promotes tumor growth by stimulating cell proliferation. Similarly, *Clostridiales* bacteria in the breast tumor tissue produce trimethylamine N-oxide (TMAO), a metabolite that activates CD8+ T cells and promotes anti-tumor immunity [137,139].

## 5. Harnessing Microbes for Immunotherapy Success

One potential method for treating several malignancies, including breast cancer, is cancer immunotherapy [140]. Influencing the immunological responses of the body both locally and systemically is the microbiome. Studies have demonstrated that the gut microbiota can affect the effectiveness of immune checkpoint inhibitors (ICIs) and CTLA-4 antibodies, two types of cancer immunotherapies [141,142].

Research conducted on animal models has brought attention to the significance of certain gut bacteria in influencing the efficacy of immunotherapies. In mouse models, it has been demonstrated that the presence of certain Bacteroides species increases the effectiveness of CTLA-4 inhibition [143,144]. Similarly, Bifidobacterium spp., Ruminococcaceae, and Faecalibacterium have been associated with improved responses to anti-PD-1 immunotherapy in melanoma patients [145].

Tumor microenvironment and systemic immune responses can both be impacted by the gut microbiota’s makeup. An increased variety of Ruminococcaceae and Faecalibacterium in the gut microbiome, for instance, has been associated with improved anti-tumor immunity and better results for immunotherapy patients [146].

Novel approaches to treating breast cancer have been made possible by recent developments in adoptive cell treatments, such as chimeric antigen receptor (CAR) cell therapy [147]. Preclinical and clinical research on CAR-T and CAR-NK cells, which are designed to identify and combat cancer cells, have demonstrated promise [148]. However, the effectiveness of these therapies can be influenced by the patient’s microbiome, further underscoring the complex interplay between gut health and cancer treatment.

## 6. Microbial Shields: How Bacteria Influence Treatment Resistance

Chemoresistance is a major factor in treatment failure and patient death for breast cancer patients, and drug resistance is still a major obstacle in this regard [149]. The microbiome has been implicated in influencing drug resistance through various mechanisms. Chemotherapy and radiation are standard treatments for breast cancer, but their effectiveness can be modulated by the microbiota [42]. For instance, it has been demonstrated that the microbiome’s composition of certain bacteria and fungus influences how well radiation treatment works on tumors. Commensal fungi have the ability to control the immunosuppressive microenvironment, whereas bacteria are required for effective immune responses against tumors [150] (Table 4).

Moreover, microbiota can impact drug metabolism. Irinotecan, a common chemotherapy drug, is converted to its active form in the liver and subsequently processed in the gut. Gut bacteria can reactivate the drug, leading to adverse side effects such as diarrhea [151]. *Enterococcus faecalis* is known to produce a β-glucuronidase enzyme that can deconjugate irinotecan, a chemotherapy drug. This deconjugation releases the active metabolite SN-38 in the intestine, which can lead to severe gastrointestinal side effects, including diarrhea. Similarly, *Escherichia coli* and *Clostridium* species also produce β-glucuronidase enzymes, which have been implicated in the reactivation of other drugs, exacerbating their side effects. Likewise, breast cancer drugs like trastuzumab and letrozole have been shown to influence microbiota composition, which may affect treatment outcomes [152]. Treatment with trastuzumab has been observed to reduce populations of *Lactobacillus* and *Bifidobacterium*, both beneficial bacteria that are associated with anti-inflammatory effects and immune modulation. The decrease in these beneficial strains can weaken the gut’s immune–supportive environment and potentially increase susceptibility to gastrointestinal inflammation. This alteration in microbiota can impair the immune response needed to combat cancer cells, potentially impacting the overall effectiveness of trastuzumab. Letrozole treatment has been linked to an increase in certain Firmicutes species and a reduction in *Akkermansia muciniphila*, a bacterium known for its beneficial effects on gut barrier integrity and metabolic health. The reduction in *A. muciniphila* may compromise gut health and lead to systemic inflammation, which could interfere with treatment efficacy. Furthermore, an increase in the pathogenic Firmicutes species can exacerbate dysbiosis and contribute to adverse side effects, which may negatively impact patient outcomes.

## 7. Microbiome Research in Clinical Practice: Clinical Trials and Future Directions

The goal of several ongoing clinical trials is to investigate the connection between breast cancer therapy and the microbiome. These researchers are looking at a number of topics, such as how the microbiome affects chemotherapy side effects, how beneficial probiotics are, and whether or not microbiome modulation might improve treatment outcomes.

Key trials include:

- Gut Microbiome Components Predict Response to Neoadjuvant Therapy in HER2-positive Breast Cancer Patients (NCT05444647) (July 2022 to March 2024): Investigates how gut microbiome composition affects responses to neoadjuvant therapy [34].
- Exercise, Gut Microbiome, and Breast Cancer (EMBRACE) (NCT05000502) (August 2021 to January 2024): Examines the interplay between exercise, gut microbiome, and breast cancer outcomes [40].
- Assessing the Impact of the Microbiome on Breast Cancer Radiotherapy Toxicity (NCT04245150) (March 2019 to July 2024): Focuses on how microbiome affects radiotherapy-related side effects [40].

These trials aim to provide valuable insights into how microbiome-targeted interventions can potentially improve breast cancer treatment outcomes and quality of life for patients.

## 8. Justification for the Current Review

Our review provides new insights beyond the scope of the 2023 paper by Nandi, Parida, and Sharma [9], with a distinct emphasis on specific emerging mechanisms, therapeutic implications, and personalized medicine approaches related to the gut microbiota’s role in breast cancer.

Our review highlights emerging mechanistic pathways through which the gut microbiome influences breast cancer progression, specifically examining microbial metabolites such as short-chain fatty acids (SCFAs), bile acids, and compounds involved in estrogen metabolism [153]. Unlike the prior work, we provide a comprehensive analysis of how these metabolites modulate the immune function, tumor microenvironment, and hormone regulation, and how these interactions might serve as potential biomarkers and therapeutic targets [154]. Additionally, our review expands on the therapeutic implications of gut microbiome modulation, focusing on personalized microbiota-based treatments [155]. We analyze the latest findings from clinical trials and preclinical studies on the efficacy of probiotics, prebiotics, and dietary interventions, exploring their potential to enhance breast cancer treatments, reduce side effects, and address treatment resistance. By emphasizing microbiome profiling, our review also proposes that gut microbiota composition could be used to stratify patients and tailor therapies, an innovative approach not extensively covered in previous reviews. Lastly, we delve into the specific relationship between gut microbiota and estrogen metabolism, providing a nuanced discussion on how microbial dysbiosis influences estrogen reabsorption and hormone receptor activity. This perspective broadens the understanding of the microbiome–hormone interaction in breast cancer, particularly for hormone-dependent subtypes. By focusing on these unique areas, our review contributes a new, specialized perspective to the field, emphasizing the potential of the gut microbiota in developing more personalized and effective breast cancer treatments.

## 9. Conclusions

A challenging yet exciting new area of cancer research has been revealed by the investigation of the gut microbiota’s connection to breast cancer. The field’s therapeutic importance is further highlighted by the notion of a gut–breast microbiota axis, which is developing, and the potential use of microbiome profiles as biomarkers. Even while we now understand these interactions much better, there are still a lot of unanswered questions. Subsequent investigations ought to concentrate on clarifying certain microbial processes, pinpointing important bacterial species and metabolites, and designing focused remedies. As our understanding grows, there is significant potential to improve patient outcomes by incorporating microbiome research into programs for breast cancer prevention, detection, and therapy. This evolving field emphasizes the need for a holistic approach to cancer biology, considering the intricate ecosystem within which tumors develop and progress, and opens up new avenues for innovative therapeutic approaches.

## Figures and Tables

**Table 1:** Microbial infections and their role in global cancer burden.

Microbe	Type	Associated Cancer(s)	Geographical Association	Mechanism	Reference
*Helicobacter pylori (H. pylori)*	Bacteria	Gastric cancer, possibly linked to breast cancer	Eastern Asia (highest), Sub-Saharan Africa	Causes chronic inflammation, leading to DNA damage	[46]
*Human Papillomavirus (HPV)*	Virus	Cervical, oropharyngeal, anal cancers, potentially breast cancer	Eastern Asia, Northern Europe, West Asia	Disrupts cell cycle via oncoproteins E6 and E7, causing tumor growth	[47]
*Hepatitis B virus (HBV)*	Virus	Liver cancer	East Asia, Sub-Saharan Africa	Chronic infection leading to cirrhosis and cancer	[48]
*Hepatitis C virus (HCV)*	Virus	Liver cancer	East Asia, Europe, Americas	Persistent infection causing inflammation, cirrhosis, and tumors	[49]
Gut Microbiota	Mixed (Bacteria, Virus, Fungi)	Breast cancer (influences hormone levels, immune response)	Global distribution	Modulates hormone metabolism, immune responses, and inflammation	[40]
Local Breast Microbiota	Bacteria	Breast cancer (local microbiota in tumors vs. healthy tissue)	Global, subject to emerging research	Alters local immune environment, may promote or inhibit tumor growth	[50]

**Table 2:** Gut microbiota and estrogen in breast cancer.

Factor	Role	Effect on Breast Cancer	Reference
Hormone Therapy	Targets hormone-sensitive breast cancers	Modulates tumor growth	[88]
Gut Microbiota	Regulates estrogen via β-glucuronidase	Influences cancer risk	[72]
Dysbiosis	Imbalance in gut bacteria	Alters estrogen metabolism	[89]
Diet (Soy Products)	Provides phytoestrogens	Linked to reduced risk	[90]
Probiotics (Lactobacillus)	Supports healthy gut microbiota	May lower cancer incidence	[91]

**Table 4:** Microbiome influence on breast cancer treatment.

Aspect	Microbiome Influence	Details	References
Treatment Efficacy	Microbiome composition (bacteria and fungi) modulates effectiveness of radiation treatment	Commensal fungi control immunosuppressive microenvironment; Bacteria necessary for effective immune responses against tumors	[145]
Drug Metabolism	Gut bacteria can reactivate chemotherapy drugs	Irinotecan is converted to active form in liver, processed in gut. Bacterial reactivation can lead to side effects like diarrhea	[146]
Treatment–Microbiome Interaction	Breast cancer drugs can alter microbiota composition	Trastuzumab and letrozole shown to influence microbiota composition, potentially affecting treatment outcomes	[147]
Chemoresistance	Microbiome implicated in influencing drug resistance	Various mechanisms contribute to chemoresistance, a major factor in treatment failure and patient death	[143,144]

## Data Availability

No new data were created or analyzed in this study.
